# Optimizing the Extraction of Bioactive Compounds from *Porphyra linearis* (Rhodophyta): Evaluating Alkaline and Enzymatic Hydrolysis for Nutraceutical Applications

**DOI:** 10.3390/md22060284

**Published:** 2024-06-18

**Authors:** Débora Tomazi Pereira, Paz García-García, Nathalie Korbee, Julia Vega, Francisco J. Señoráns, Félix L. Figueroa

**Affiliations:** 1 Experimental Center Grice Hutchinson, Institute of Blue Biotechnology and Development (IBYDA), University of Malaga, Lomas de San Julián, 2, 29004 Malaga, Spain; debora.tomazi@uma.es (D.T.P.); nkorbee@uma.es (N.K.); juliavega@uma.es (J.V.); 2 Group of Bioactive Extracts and Healthy Lipids, Faculty of Sciences, Cantoblanco Campus, 28049 Madrid, Spain; mpgarciagarcia@hotmail.com (P.G.-G.); javier.senorans@uam.es (F.J.S.)

**Keywords:** alkaline hydrolysis, biological active compounds, bioeconomics, biorefining, enzymes, extraction methods, *Porphyra*, nutraceuticals

## Abstract

*Porphyra sensu lato* is one of the most economically significant and widely cultured and consumed algae in the world. *Porphyra* species present excellent nutraceutic properties due to their bioactive compounds (BACs). This research aimed to find the most efficient aqueous extraction method for BACs by examining alkaline and enzymatic hydrolysis. Alkaline hydrolysis with 2.5% sodium carbonate (SC) and at 80 °C proved optimal for extracting all BACs (phycobiliproteins, soluble proteins, polyphenols, and carbohydrates) except mycosporine-like amino acids (MAAs), which were best extracted with water only, and at 80 °C. Enzymatic hydrolysis, particularly with the ‘Miura’ enzymatic cocktail (cellulase, xylanase, glycoside hydrolase, and β-glucanase), showed superior results in extracting phycoerythrin (PE), phycocyanin (PC), soluble proteins, and carbohydrates, with increases of approximately 195%, 510%, 890%, and 65%, respectively, compared to the best alkaline hydrolysis extraction (2.5% SC and 80 °C). Phenolic content analysis showed no significant difference between the ‘Miura’ cocktail and 2.5% SC treatments. Antioxidant activity was higher in samples from alkaline hydrolysis, while extraction of MAAs showed no significant difference between water-only and ‘Miura’ treatments. The study concludes that enzymatic hydrolysis improves the efficiency of BACs extraction in *P. linearis*, highlighting its potential for the nutraceutical industry, and especially with respect to MAAs for topical and oral UV-photoprotectors.

## 1. Introduction

The red macroalgae *Porphyra sensu lato* is one of the most economically significant and widely cultured and consumed algae in the world [1,2,3,4]. This is largely because it is one of the highest producers of bioactive compounds (BACs) [5], including biliproteins, polyphenols, proteins, polysaccharides, and mycosporine-like amino acids (MAAs), all of which positively impact human health [6].

Biliproteins are a group of water-soluble proteins, categorized as secondary metabolites, that have an associated chromophore which is responsible for light-harvesting [7]. Studies on phycobiliproteins have demonstrated the potential of these compounds in various therapeutic applications, including the demonstration of antioxidant [8,9,10], antiviral [10,11,12], anticancer [13,14], antimicrobial [10], antidiabetic [15], anti-inflammatory [16,17], immunomodulatory [18], neuroprotective [19], and hepatoprotective [16,20] effects.

Polyphenols are secondary metabolites that commonly possess at least one benzene ring attached to the hydroxyl group(s) [21]. They play a significant role in the physiological processes of plants and algae, being involved in protection against UV radiation, anti-herbivory defense, resistance to pathogens, and the defense against the growth of epiphytes [22]. Polyphenols’ extracts also showed anti-inflammatory [23], antioxidant [24,25,26], anticancer [27], and anti-diabetic [26] properties.

Proteins are macromolecules, considered primary metabolites since they perform essential functions for plants and algae, such as structuring, signaling for growth and cell division, and substance transport, among others. A soluble protein extract may contain not only phycobiliproteins but also antioxidant enzymes like catalase, superoxide dismutase, and peroxidases [28], all of which confer significant antioxidant power.

In algae, polysaccharides are primary metabolites because they provide growth and structure (in the case of cellulose, provided by mannose and xylose), as well as forms of energy storage, such as starch [29]. In addition to these neutral polysaccharides, algae can also have sulfated polysaccharides, such as porphyran, in cases of algae belonging to the genus *Porphyra sensu lato*. Polysaccharides derived from *Porphyra* exhibit a wide range of physiological activities, including antioxidant [30,31,32,33], antiviral [34], immunoregulatory [32,35], anticancer [18,36], liver protection [37], anti-inflammatory [38,39], and prebiotic activities [40].

The MAAs are secondary metabolites, compounds which are nitrogenous, of low molecular weight, water-soluble and UV-absorbing [41,42]. The main function of MAAs is their photoprotective ability, described as being “microbial sunscreens” [43,44,45]. In addition, MAAs’ molecules act as antioxidants [46,47,48,49,50] and demonstrate anticancer [51,52], anti-photoaging [50,53,54,55], wound-healing [56], and anti-inflammatory [48,51] effects. MAAs are being explored as a new generation of environmentally friendly sunscreens [57,58,59].

Given all the properties that *Porphyra*’s BACs can exhibit, and due to the possibly synergistic effects that these molecules could express, this alga has become an important subject of study and interest for different industries, especially for nutricosmeceutical applications. Therefore, the aim of this research was to explore and identify the most efficient aqueous extraction method for BACs. The focus was on developing a methodology which involves testing alkaline and enzymatic hydrolysis, aiming to destabilize the cell wall and facilitate the extraction of cytoplasmic compounds, and that not only maximizes efficiency but also ensures high yields of these valuable compounds. This pursuit was driven by the growing demand for natural and bioactive ingredients across various industries, areas in which BACs have the potential to make a significant impact, whether in the formulation of skincare products, pharmaceutical applications or biotechnological advancements, or as functional ingredients in the food sector.

## 2. Results

### 2.1. Response Surface Model

After performing the extractions following the DOE and analyzing through use of the RSM, the equation for achieving optimal soluble protein extraction was determined:

Proteins = −36.0475 + 2.4123 × Temperature + 0.3686 × Time + 1.45208 × Enzyme − 0.0307 × Temperature^2^ − 0.0017 × Time^2^ − 0.0186 × Enzyme^2^ − 0.0016 × Temperature × Time − 0.0146 × Temperature × Enzyme + 0.0023 × Time × Enzyme (R^2^ = 0.96)

**Figure 1:**
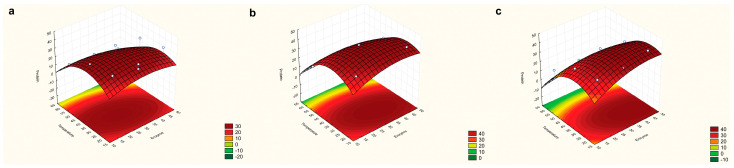
Response surface graphs associated with the contents of soluble proteins (mg·g^−1^), considering temperature (°C) and enzyme concentration (mg·g^−1^) with different times of extraction: 30 min (**a**), 105 min (**b**), and 180 min (**c**).

The optimal conditions, which were used in the extraction by enzymatic hydrolysis, were identified as 122 min, 27.66 °C, and 36 mg of enzyme per g of dry biomass (Figure 1). 

### 2.2. Effects on the Quantification of Photosynthetic Pigments: Phycobiliproteins

In the process of the extraction of BACs through alkaline hydrolysis, the phycoerythrin (PE) pigment exhibited the best extractions when water was used, at both temperatures, and with 1%, 2.5%, and 5.25% SC, all at 80 °C. The lowest PE content was obtained with extractions at 5.25% SC at 45 °C, and with 10.5% SC, at both 45 °C and 80 °C. Additionally, the highest quantity of phycocyanin (PC) was observed in samples extracted with 5.25% sodium carbonate (SC) at 80 °C and 2.5% SC at the same temperature, whereas samples extracted with 5.25% SC at 45 °C and 10.5% SC at 45 °C showed the least-favorable extractions (Table 1). 

In the alkaline hydrolysis, a positive Pearson correlation was noted between both PE and PC quantity and temperature (r = 0.4518, *p* = 0.012 for PE; r = 0.5773, *p* = 0.001 for PC). Conversely, a negative correlation was observed between PE content and sodium carbonate (SC) concentration (r = −0.6513, *p* = 0.001). Statistically, temperature presented the greatest effect on PC concentration [η^2^ = 33.33%, F_(1,20)_ = 35.233, *p* ≤ 0.001]. For PE, the SC concentration was found to have the most significant effect [η^2^ = 50.45%, F_(4,20)_ = 30.638, *p* ≤ 0.001].

In the enzymatic hydrolysis used for the extraction of BACs, the most efficient extraction, statistically speaking, of PC and PE occurred with the ‘Miura’ cocktail, which resulted in an increase of approximately 232% in the extraction efficiency of PE, and 105% for PC (Table 1).

### 2.3. Effects on the Quantification of Polyphenols, Proteins, and Carbohydrates

The best extraction of soluble phenols in alkaline hydrolysis was observed with 1% and 2.5% SC, both at 80 °C. Conversely, the least favorable extraction occurred with water at 45 °C; 1%, 2.5%, 5.25%, and 10.5% SC, all at 45 °C; and 10.5% at 80 °C. As for total soluble proteins, the highest contents in alkaline hydrolysis were observed with 1% and 2.5% SC at 80 °C, while the lowest contents were with water at 80 °C, and 1% and 10.5% SC at 45 °C. Regarding total soluble carbohydrates, the best extraction in alkaline hydrolysis was noted with 2.5% and 5.25% SC at 80 °C, whereas the least favorable was with water, at both temperatures (Table 2). 

In the alkaline hydrolysis, a positive and strong Pearson correlation was observed between temperature and the quantities of phenolics, proteins, and carbohydrates (r = 0.7123, *p* = 0.001; r = 0.3987, *p* = 0.029; and r = 0.5716, *p* = 0.001, respectively). However, no correlation was observed between these BACs and SC concentration. The temperature exhibited the most pronounced impact on phenolic content [η^2^ = 42.46%, F_(1,20)_ = 42.023, *p* ≤ 0.001]. For proteins and carbohydrates, the interaction between the factors (SC concentration and temperature) demonstrated a more substantial effect: [η^2^ = 45.57%, F_(4,20)_ = 40.781, *p* ≤ 0.001], [η^2^ = 38.63%, F_(4,20)_ = 23.417, *p* ≤ 0.001], respectively.

In enzymatic hydrolysis, the most efficient extraction of phenolics, proteins, and carbohydrates was achieved with the ‘Miura’ cocktail, which resulted in a statistically significant increase of approximately 70%, 130%, and 118%, respectively, when compared to the control (Table 2).

### 2.4. Effects on the Profiles of Mycosporine-like Amino Acids (MAAs)

In the alkaline hydrolysis, four types of mycosporine were identified by HPLC: palythine, asterina-330, shinorine, and porphyra-334. For palythine, the only extraction capable of detection was with water at 80 °C and with 2.5% SC at 45 °C, without there being significant difference between them. Asterina-330 was observed only in the extraction with 2.5% SC at 45 °C. Shinorine was detected both in water extractions and all SC concentrations, but only at 45 °C. Both water extractions and those with 1% SC showed, statistically, the highest shinorine content, whereas 2.5%, 5.25%, and 10.5% SC showed the lowest. Porphyra-334, the most abundant mycosporine detected, was not detected in some extractions with 2.5%, 5.25%, and 10.5% SC, specifically, those at 80 °C. Statistically, the best extraction of total MAAs occurred in water at 80 °C, while the least-favorable extractions occurred with 1%, 5.25%, and 10.5% SC at 45 °C (Table 3).

As for the alkaline hydrolysis, palythine, shinorine, and porphyra-334 exhibited negative Pearson correlations with SC concentration (r = −0.4753, *p* = 0.012; r = −0.5622, *p* = 0.002; and r = −0.5882, *p* = 0.001, respectively). On the other hand, no correlation was observed between each of these MAAs and temperature. The SC-concentration factor was found to be the most influential with regard to the concentration of all identified and extracted MAAs: [η^2^ = 55.51%, F_(4,20)_ = 51.3405, *p* ≤ 0.001], [η^2^ = 43.64%, F_(4,20)_ = 120.7713, *p* ≤ 0.001], [η^2^ = 89.49%, F_(4,20)_ = 522.7694, *p* ≤ 0.001], and [η^2^ = 76.91%, F_(4,20)_ = 545.422, *p* ≤ 0.001], respectively, for palythine, asterine-330, shinorine, and Porphyra-334.

In the enzymatic hydrolysis process, porphyra-334 was the only MAA identified using HPLC. The extraction that yielded the highest efficiency for this MAA was found to be the ‘Miura’ cocktail. This method led to an increase of about 31% in the extraction efficiency of porphyra-334 compared to the control, a difference that was statistically significant (Table 3).

### 2.5. Effects on Antioxidant Activity (ABTS Assay)

Considering the alkaline hydrolysis, the best antioxidant activity, statistically, was observed in the extracts obtained with 1%, 2.5%, and 10.5% SC, all at 80 °C. In opposition, the least-favorable antioxidant activity was observed in both of the extracts using only water (Table 4). A positive Pearson correlation was noted between the antioxidant activity and SC concentration (r = 0.5508, *p* = 0.002), as well as temperature (r = 0.4470, *p* = 0.013). A positive Pearson correlation was also observed between the antioxidant activity and the sum of all BACs measured above (r = 0.5465, *p* = 0.002). The SC-concentration factor exerts the most significant influence on the antioxidant response [η^2^ = 64.80%, F_(4,20)_ = 117.029, *p* ≤ 0.001] in the alkaline hydrolysis.

Regarding the enzymatic hydrolysis, the extract provided by the ‘Miura’ cocktail exhibited the highest antioxidant activity, compared with the control, showing an approximate increase of 35%, a difference that was statistically significant (Table 4).

### 2.6. Comparison between the Optimal Extractions from Alkaline and Enzymatic Hydrolysis Tests

Considering alkaline hydrolysis, the optimal overall extraction for all BACs, excluding MAAs, was observed using 2.5% SC at 80 °C. In contrast, the most effective method for extracting MAAs was that using only water, and at 80 °C. Regarding enzymatic hydrolysis, the inclusion of the ‘Miura’ enzymatic cocktail yielded better results compared to the absence of enzymes. Statistical comparison of all results within these groups revealed that the content levels of PE, PC, proteins, and carbohydrates were considerably higher in samples treated with ‘Miura’, showing increases of approximately 195%, 510%, 890%, and 65%, respectively. However, the phenolic content did not significantly differ between the ‘Miura’ cocktail and that of 2.5% SC at 80 °C. On the other hand, antioxidant activity was notably higher (by 285%) in samples derived from alkaline hydrolysis, compared to those from enzymatic hydrolysis. In terms of the total quantity of MAAs, there was no statistically significant difference observed between samples extracted with water at 80 °C and those extracted with the ‘Miura’ cocktail (Table 5).

### 2.7. Comparison between the Optimal Extractions from the Present Study and other References with Good Extraction Results for PE, Phenols, and MAAs 

The study compared the optimal extractions for PE, phenols, and MAAs (the three most important antioxidant compounds in *P. linearis*) in alkaline and enzymatic hydrolysis with other references involving *Porphyra* species and considered the concentration of each BAC achieved, the extraction solvent, the ‘Globally Harmonized System of Classification and Labeling of Chemicals’ (GHS) in terms of acute toxicity, the protocol, and the costs. Regarding the PE concentration, the best result in the present study was observed with the presence of the ‘Miura’ cocktail. The best result for phenols was observed in other protocols using methanol or acetone, though these increased the levels of toxicity. Considering the content levels of MAAs, the best results were observed with either only water or the ‘Miura’ cocktail, both at 80 °C, as found in this present work (Table 6).

### 2.8. Principal Component Analysis and Analyses of BACs from P. linearis Extracts Based on Alkaline and Enzymatic Hydrolysis Tests

**Figure 2:**
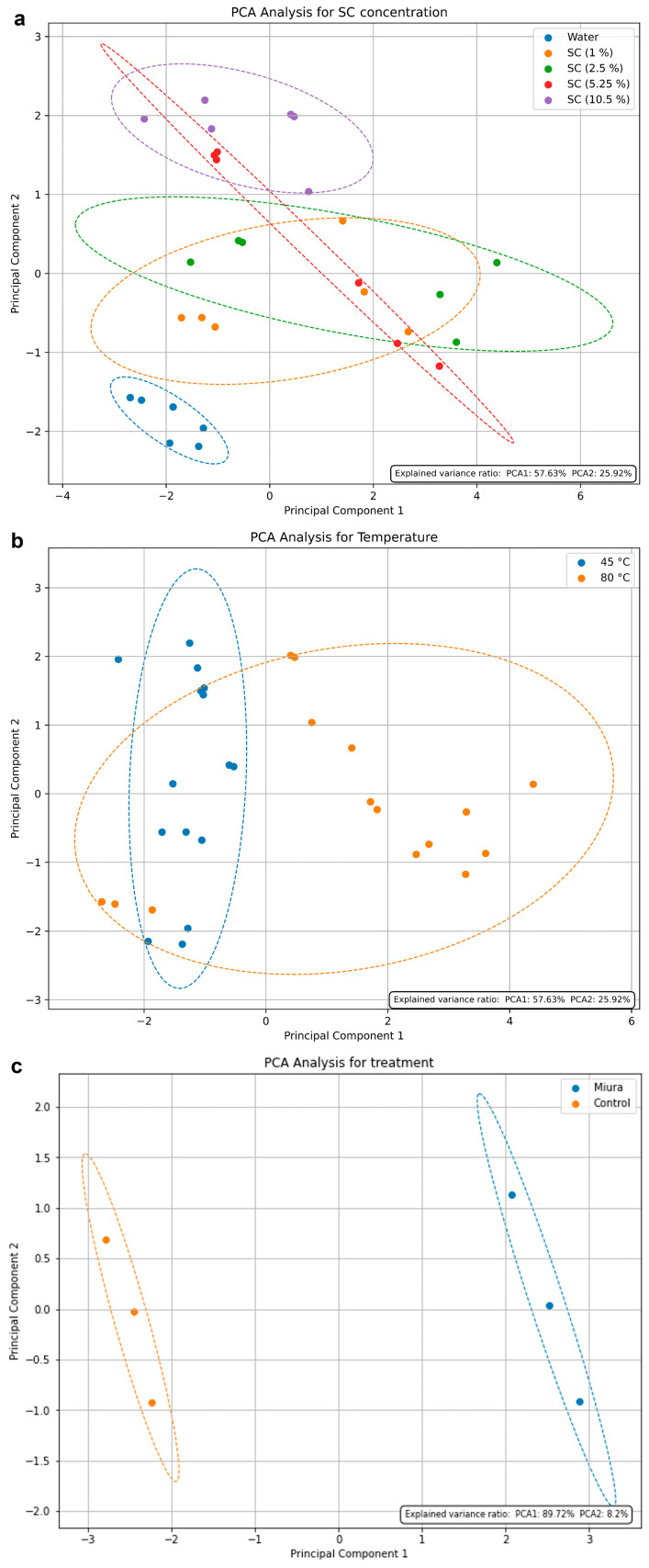
Principal component analysis (PCA) of BACs of *P. linearis* (PE, PC, total soluble phenolics, proteins, carbohydrates, total MAAs, and antioxidant activity (ABTS)), considering both sodium carbonate concentration (**a**) and temperature (**b**) in alkaline hydrolysis, and the type of treatment (control and ‘Miura’) in enzymatic hydrolysis (**c**).

The PC1 and PC2 explained 83.55% of the variation in the samples obtained from alkaline hydrolysis. Considering the SC concentration, a completely displaced group is observed for the samples extracted with water only, and a slight displacement for the samples extracted with 10.5% SC (Figure 2a). On the other hand, it can be observed that the samples from water-only extraction show less dispersion among themselves, while the presence of SC causes elevated dispersion among the samples. This can be understood by taking into account the temperature. Excluding the water-only extracted samples, the temperature PCA shows the noticeable formation of two distinct groups (Figure 2b). This highlights the fact that temperature does not cause variation in the water-only extractions, whereas the extraction with SC is altered by the temperature.

In the case of enzymatic hydrolysis, PC1 and PC2 accounted for 97.92% of the variation (with PC1 alone accounting for almost 90%), clearly showing the formation of distinct groups among samples derived from extraction with the ‘Miura’ cocktail and those from extraction without enzymes (control). The significant distance between the two groups suggests significant differences between them, whereas the control samples are a bit more clustered together, showing that they are more homogeneous, compared to the ‘Miura’ samples (Figure 2c).

**Table 6:** Concentrations of phycoerythrin (PE), phenolics, and total MAAs (mg·g^−1^ dry weight) in *P. linearis* and other references associated with *Porphyra* species, considering the extraction solvent, the level of acute toxicity based on the ‘Globally Harmonized System of Classification and Labelling of Chemicals’ (GHS), the protocol, and the costs.

Compound	Specie	Concentration (mg·g^−1^)	Extraction Solvent	GHS (Safety)	Protocol	Costs	Reference
**PE**	*Porphyra linearis*	4.12 ± 0.45	Sodium carbonate 2.5% in water	0	1.5 h at 80 °C	+	present study
	*Porphyra linearis*	12.20 ± 3.70	‘Miura’ cocktail in sodium citrate buffer 0.1 M, pH 4.5	0	2 h at 28 °C	++	present study
	*Porphyra* spp.	8.32 ± 0.29	Phosphate buffer (pH = 6.8)	0	15 min of centrifugation	+	[60]
	*Porphyra leucosticta*	6.11 ± 0.39	N,N-dimethylformamide	4	24 h at 4 °C	+	[61]
**Polyphenols**	*Porphyra linearis*	3.53 ± 0.64	Sodium carbonate 2.5% in water	0	1.5 h at 80 °C	+	present study
	*Porphyra linearis*	3.08 ± 0.22	‘Miura’ cocktail in sodium citrate buffer 0.1 M, pH 4.5	0	2 h at 28 °C	++	present study
	*Pyropia acanthophora* var. *brasiliensis*	9.09 ± 0.34	80% Methanol	3	1 h at room temperature	++	[62]
	*Porphyra umbilicalis*	15.50 ± 0.50	70% Acetone	3	24 h at 4 °C	++	[63]
**MAAs**	*Porphyra linearis*	20.25 ± 4.80	Water	0	1.5 h at 80 °C	+	present study
	*Porphyra linearis*	20.75 ± 0.05	‘Miura’ cocktail in sodium citrate buffer 0.1 M, pH 4.5	0	2 h at 28 °C	++	present study
	*Porphyra umbilicalis*	approx. 10.00	20% Methanol	3	2 h at 45 °C and 10 min of centrifugation at 4 °C, evaporated in a vacuum centrifuge for 24 h, and redissolved in 100% methanol	+++	[64]
	*Porphyra umbilicalis*	approx. 15.00	20% Methanol	3	2.5 h at 45 °C, redissolved in 100% methanol, evaporated to dryness at 45 °C, and redissolved in distilled water	+++	[65]
	*Porphyra columbina*	10.60	20% Methanol	3	2 h at 45 °C and 10 min of centrifugation at 4 °C, evaporated in a vacuum centrifuge for 24 h, and redissolved in 100% methanol	+++	[66]
	*Porphyra haitanensis*	approx. 8.00	100% Methanol	3	24 h at 4 °C	++	[67]
	*Pyropia columbina*	10.40 ± 1.10	20% Methanol	3	2.5 h at 45 °C, redissolved in 100% methanol, evaporated to dryness at 45 °C, and redissolved in distilled water	+++	[68]
	*Pyropia acanthophora*	6.65	20% Methanol	3	2 h at 45 °C and 10 min of centrifugation at 4 °C, evaporated in a vacuum centrifuge for 24 h, and redissolved in 100% methanol	+++	[69]
	*Porphyra umbilicalis*	5.20 ± 0.40	Water	3	Extracts dried under vacuum, and re-suspended in 100% methanol	++	[70]

+: low cost; ++: medium cost; +++: high cost.

## 3. Discussion

Algae belonging to the group *Porphyra sensu lato* consist of only a single-cell layer, surrounded by a thick cell wall which provides protection and support to these cells in stress conditions in the intertidal system. This cell wall is composed of cellulose, xylose, mannose, galactose, porphyran, arabinose, and glucose [3,71,72,73,74]. Therefore, to extract the economically valuable BACs located in the cytoplasm or within organelles, it is necessary to breach this robust structure surrounding the cell. There are two main groups of cell disruption processes: mechanical–physical processes, such as grinding, sonication, pulsed electric, microwaves, freezing and thawing, and heating; and (bio)chemical processes, including alkaline hydrolysis, acid hydrolysis, and enzymatic hydrolysis [75,76,77]. This study compared the use of two combinations of these processes: grinding, heating, and alkaline hydrolysis; and grinding, heating, and enzymatic hydrolysis. It confirms that new methodologies for sustainable, cost-effective, and efficient extractions are achievable. The search for more-efficient extractions of the BACs with significant economic interest not only aids in enhancing extraction efficiency but also in the optimal use of biomass, respecting the living organism from which the compounds are derived, and requiring less biomass production, as more compounds are extracted using less biomass. Furthermore, improved extraction techniques can lead to reduced production costs, requiring less physical space and lower water consumption, especially in the case of algae production.

Within the pigments known as phycobiliproteins, phycoerythrin is the most exposed pigment within the antennae complex, with phycocyanin being located just beneath it and thus a bit more protected [78]. Phycoerythrin was best-extracted using only water, at both tested temperatures, demonstrating that its more exposed location within the chloroplasts facilitates its extraction. On the other hand, the use of varying concentrations of sodium carbonate (2.5% and 5.25%, both at 80 °C), also showed good extraction efficiency. The increase in pH due to alkaline hydrolysis weakness the cell wall components, because the OH- ion binds to the anomeric carbon of carbohydrate molecules, causing the cleavage of the ether bond, resulting in the formation of smaller molecules [79,80], but a higher temperature is required to complete this extraction. For the phycocyanin pigment, alkaline hydrolysis with 1%, 2.5%, and 5.25% sodium carbonate demonstrated the best extraction, but again, a higher temperature was necessary. The higher concentration of sodium carbonate (10.5%) led to a significant increase in the pH of the aqueous solution (reaching about 11–12 by the end of the extraction), which resulted in the degradation of wall carbohydrates, but, on the other hand, it may affect the phycobiliproteins’ conformation, leading to their dissociation [81]. The presence of a ‘Miura’ cocktail of enzymes specific to the cell wall of *P. linearis* breaks the bonds between carbohydrate molecules, resulting in the degradation and disintegration of the cell wall, thereby creating openings in this cellular structure and facilitating the penetration of the extraction solvent into the cytoplasm. Thus, with enzymatic hydrolysis, significantly higher amounts of phycocyanin and phycoerythrin were extracted compared to the control extraction, but also as compared to the best extraction obtained by alkaline hydrolysis (2.5% at 80 °C). The acidic pH of the extraction solvent in enzymatic hydrolysis, along with constant agitation due to the incubator, may have facilitated the extraction of these compounds. High concentrations of phycoerythrin in untreated thalli were observed in *Porphyra* sp. by [60] at 8.319 mg·g^−1^, and in *Porphyra leucosticta* by [61] at 6.11 mg·g^−1^, in addition to phycocyanin in *Porphyra* sp. by [61] at 5.305 mg·g^−1^ and *Porphyra umbilicalis* by [82] at 4.9 mg·g^−1^. The present work achieved levels of 12.20 and 6.71 mg·g^−1^ for phycoerythrin and phycocyanin, respectively.

Total soluble proteins also showed optimal concentrations when extracted with the ‘Miura’ cocktail, compared to alkaline hydrolysis, since the surface response model was calculated, taking into account the protein results. There was an increase of approximately 890%, reaching a concentration of total soluble proteins of 55.63 mg·g^−1^. Reference [61] obtained results of 44.7 mg·g^−1^ in *P. leucosticta* and 23.62 mg·g^−1^ in *P. umbilicalis* [83], which highlights the fact that we extracted a higher concentration, when compared with other species of *Porphyra*, using the hydrolysis technique.

Soluble carbohydrates also showed better extraction when the algae were subjected to enzymatic hydrolysis. Since the enzymes used in the ‘Miura’ cocktail are specifically employed to break down the cell-wall polysaccharides of *Porphyra*, the final extraction of these compounds is facilitated, as the enzymes also acted as an extractor. It is known that the higher the temperature used in the extraction process, the more carbohydrates are extracted [84,85]. Even when using a low temperature in the enzymatic hydrolysis (28 °C), as compared to the temperature used in alkaline hydrolysis (80 °C), an excellent result was achieved, with an increase of 65% resulting; this also increased the preservation of the compounds of interest and reduced the energy expenditure.

The final concentration of soluble phenols and the total MAAs did not show differences when extracted via alkaline or enzymatic hydrolysis. The main phenols identified in *Porphyra sensu lato* include catechins, epicatechins, gallic acid, 4-hydroxybenzoic acid, epigallocatechin, epigallocatechin gallate, salicylic acid, rutin, and hesperidin [23,86,87,88], which are polar compounds. Thus, they can be extracted with water, or more commonly with 80% methanol [62] or 70% acetone [64]. Methanol and acetone, being slightly less polar than water, are particularly effective at extracting a wide range of phenol compounds, including those that are slightly less polar but not entirely non-polar. This explains the higher concentrations of phenols found in *Porphyra* sp. by [63] (15 mg·g^−1^), in *P. umbilicalis* by [64] (16 mg·g^−1^), and in *Pyropia acanthophora* var. *brasiliensis* by [62] (9.09 mg·g^−1^), as compared to the findings in the current study in *P. linearis* of 3.53 mg·g^−1^. In the present research, we utilized water extraction as a technique to avoid the toxicity of solvents such as methanol or acetone, in order to provide biosecurity for the food industry.

MAAs were more effectively extracted with the presence of the ‘Miura’ cocktail through enzymatic hydrolysis, and during the alkaline hydrolysis tests, only with the water at 80 °C. It is known that an increase in pH can cause the degradation and destabilization of some molecules, such as MAAs [89], explaining the reason for the absence or low extraction levels of these compounds in the presence of sodium carbonate. High photo- and thermostability of MAAs, mainly under low basic and acid solutions, has been previously reported [89,90,91,92]. Furthermore, this study highlights that the interaction between the presence of carbonate and high temperatures becomes stronger and causes this degradation. In both optimal extractions, a total of approximately 20 mg·g^−1^ was achieved; this level is significantly high compared to those previously described for *Porphyra* and *Pyropia* spp. from different coastal areas of Europe, China, and South America, ranging from 5 to 15 mg·g^−1^ [64,65,66,67,68,69,70]. Only in other species of Bangiales, such as the *Bangia atropurpurea* collected in South Island, New Zealand and cultivated for 6 months in Provasoli-enriched North Sea water [93] under white light [94], have total MAAs extracted in 25% aqueous methanol (*v*/*v*) at 45 °C reached values close to 19 mg·g^−1^ DW, as in this study in *P. linearis*.

The antioxidant activity test with ABTS demonstrates the capacity of antioxidant agents present in the extract to neutralize free radicals. The best extractions of the antioxidant compounds were obtained with 1% and 2.5% sodium carbonate at 80 °C, which also exhibited the best antioxidant activity. The extracts from the extraction with 10.5% sodium carbonate at 80 °C showed good antioxidant activity as well, which may be due to the presence of a high concentration of total carbohydrates, which could include a significant portion of sulfated carbohydrates with high antioxidant activity, or due to the presence of some other oxidizing agent not quantified in this work, such as ascorbic acid [95,96]. The presence of the enzymatic cocktail improved the antioxidant activity of this extract, when compared to its control. On the other hand, when comparing between the hydrolyses, the antioxidant response was superior in the extract from alkaline hydrolysis, even though the extract from enzymatic hydrolysis had higher concentrations of most compounds. The precipitation of the enzymes used for enzymatic hydrolysis was not performed at the end of the extraction, as precipitating these enzymes would also precipitate our compounds of interest. Therefore, the presence of these commercial enzymes might be affecting the antioxidant response in some way.

The generation of PCAs demonstrates that the presence of sodium carbonate in the extraction solvent produces similar extracts, while higher concentrations of the alkaline salt (10.5%) or its absence (water-only) result in more-distinct extracts. Temperature, in the alkaline hydrolysis process, is also a crucial factor for extraction, and it was observed that higher temperatures favor the extractions of all analyzed compounds. The PCA analysis for enzymatic hydrolysis confirms that the presence of the ‘Miura’ cocktail is crucial for efficient extraction.

## 4. Materials and Methods

### 4.1. Biological Material

The gametophytes of *P. linearis* Greville were collected in the upper littoral area, on rocky shores completely exposed to the air during low tide, of Santa Cristina Beach (43°61′ N and 8°18′ W; salinity of 32 psu; spring temperature 15 °C; transparency of the coastal water type I; 0.7 mmol·m^−3^ of NO_3_^−^; 0.02 mmol·m^−3^ of PO_4_^−^; 263.26 mmol·m^−3^ of O_2_ (‘My Ocean Pro—Copernicus Marine Open Data’ platform)), Galicia, Spain, in May 2023, by the company ‘Porto Muiños’. Algal thalli were transported to the Institute of Blue Biotechnology and Development (IBYDA) of Malaga University (Spain), in plastic containers containing seawater inside of a thermal box. In the laboratory, the thalli were washed with diluted artificial seawater and screened for removal of the contaminants. Healthy portions were selected and frozen at −80 °C for future extractions of the bioactive compounds (BACs).

### 4.2. Alkaline Hydrolysis

The extraction of BACs by alkaline hydrolysis was performed at the IBYDA. In a test tube, 750 mg of fresh algae were mixed with 7.5 mL of extraction solvent. The extraction involved the use of distilled water and distilled water with varying concentrations of sodium carbonate (SC) (1%, 2.5%, 5.25%, and 10.5%) for alkaline hydrolysis. After combining the algae with their respective extraction solvents, they were macerated using an UltraTurrax^®^ (T25, IKA, Staufen, Germany) (18,000 rpm, 30 s) and then subjected to extraction. This process was conducted at two different temperatures (45 °C and 80 °C) for 1.5 h. In total, 10 different types of extractions were obtained (*n* = 3). These two temperatures were selected because 45 °C is the temperature determined by the methodology for extracting mycosporine-like amino acids [97], and it was thought interesting to test a higher temperature, since it is known that high temperatures degrade cell wall polysaccharides, facilitating the extraction of internal compounds. Additionally, 80 °C produces a thermic shock without the expectation high degradation of the most BACs. Post-extraction, the samples were centrifuged at 2721.6× *g*, and the supernatant was collected. The pH was measured using a Horiba LaquaTwin pH meter and adjusted to a pH of 7.0 using lactic acid, in a method similar to the water-only extraction. Consequently, the algal extracts were prepared for the subsequent measurement of the concentrations of BACs and antioxidant activity.

### 4.3. Enzymatic Hydrolysis

The extraction of BACs by enzymatic hydrolysis was conducted at the ‘Healthy-Lipids Group’ of the Autonomous University of Madrid, Spain. In a Falcon tube, 1.25 g of fresh algae were mixed with 10 mL of extraction solvent (Sodium Citrate Buffer 0.1 M, pH 4.5, plus an enzyme cocktail). The enzyme cocktail, named ‘Miura’, is composed of three commercial enzymes in equal proportions, Celluclast 1.5L^®^ (cellulase), Shearzyme 2x^®^ (xylanase and glycoside hydrolase), and Ultimase BWL^®^ (β-glucanase, xylanase and cellulase), generously provided by the company ‘Novozymes’ (Bagsværd, Denmark). After combining the algae with the extraction solvent (*n* = 3), they were macerated using a high-speed homogenizer (FSH-2A, Vevor^®^, Shanghai, China) (18,000 rpm) for 30 s and then subjected to extraction in an incubator with agitation (326 rpm); time, temperature and enzymatic concentration were determined by surface response model (RSM) experiment. Post-extraction, the samples were centrifuged at 2721.6× *g* at room temperature, and the supernatant was collected. Subsequently, 10 mL of distilled water was added to the biomass for a second extraction, using the homogenizer, at 18,000 rpm for 5 min. The samples were then centrifuged at the same speed and at room temperature, and the supernatant was collected. The two supernatants were stored separately. The pH was measured using a Horiba LaquaTwin pH meter and adjusted to a pH of 7.0 using sodium hydroxide, similarly to the water-only and alkaline hydrolysis extractions. Consequently, the algal extracts were prepared for the subsequent measurements of the concentrations of BACs and antioxidant activity levels.

### 4.4. Determination of Bioactive Compounds (BACs)

#### 4.4.1. Phycobiliproteins

The determinations of phycobiliprotein concentrations [phycocyanin (PC), and phycoerythrin (PE)] were performed using a UV–visible spectrophotometer (Shimadzu UV-2600, λ = 498, 615, and 651 nm, Kyoto, Japan), following the formulas described by [98]. The analyses were conducted in triplicate, and the results were expressed in mg of pigments per g of dry weight (DW).

#### 4.4.2. Soluble Proteins

The analysis of soluble proteins was carried out using the spectrophotometric Bradford method, based on [99]. Aliquots of 50 µL from the algal extracts were added to 750 µL phosphate buffer (0.1 M, pH 6.5) and 200 µL of the Bradford reagent (BioRad, Fort Worth, TX, USA) and incubated for 15 min at room temperature. Subsequently, readings were taken at 595 nm using a UV–visible spectrophotometer. The quantification of total proteins was determined using a standard curve of bovine albumin (Sigma-Aldrich, Steinheim, Germany) (4 to 60 µg·mL^−1^ − R^2^ = 0.99; y = 0.0244x; where y represents absorbance and x represents concentration). The analyses were performed in triplicate, and the results were expressed in mg of bovine albumin per g of DW.

#### 4.4.3. Soluble Polyphenols

The analysis of phenolic compounds was carried out using the spectrophotometric Folin–Ciocalteu method, based on [100]. Aliquots of 100 µL from the algal extracts were added to 700 µL distilled water, 50 µL of the Folin–Ciocalteu reagent (Sigma-Aldrich), and 150 µL of 20% sodium carbonate, and incubated for 2 h at 4 °C. Subsequently, readings were taken at 760 nm using a UV–visible spectrophotometer. The quantification of total phenolic compounds was determined using a standard curve of phloroglucinol (1 to 20 µg·mL^−1^ − R^2^ = 0.99; y = 0.0586x; where y represents absorbance and x represents concentration). The analyses were performed in triplicate, and the results were expressed in mg of phloroglucinol per g of DW.

#### 4.4.4. Soluble Carbohydrates

The analysis of carbohydrates was carried out using the spectrophotometric method, based on [101]. Aliquots of 500 µL from the algal extracts were added to 1 mL anthrone 0.2% (*w*/*v*). The samples were subjected to a dry bath at 100 °C for 3 min to carry out the reaction and were subsequently read on a UV–visible spectrophotometer at 630 nm. The quantification of soluble carbohydrates was determined using a standard curve of glucose (25 to 200 µg·mL^−1^ − R^2^ = 0.99; y = 0.01109x; where y represents absorbance and x represents concentration). The analyses were performed in triplicate, and the results were expressed in mg of glucose per g of DW.

#### 4.4.5. Mycosporine-like Amino Acids (MAAs) 

The determinations of the concentrations of MAAs were carried out according to [96], with the modifications of [102]. A quantity of 500 μL of algal extracts was filtered through a 0.2 µm membrane before chromatographic analysis using a HPLC system (1260 Agilent InfinityLab Series, Santa Clara, CA, USA) with a Diode-Array Detection (DAD) detector. The separation of the MAAs was performed by injecting 10 µL of algal extract into a C8 Luna Column (250 mm length and 4.6 mm diameter, and 5 µm particle size; Phenomenex, Aschaffenburg, Germany) which was kept at 20 °C, and the samples were maintained at 10 °C, using an isocratic run containing 1.5% aqueous methanol (*v*/*v*) plus 0.15% acetic acid (*v*/*v*) in MiliQ water as the mobile phase, with a flow rate of 0.5 mL·min^−1^; each run took 30 min. MAAs were detected at 320 and 330 nm. Isolated MAAs determined through High Performance Countercurrent Chromatography (HPCCC) were used as standards [103]. The quantification was performed using published molar extinction coefficients (ε) of the different MAAs [104,105]; the results were expressed in mg per g of DW.

### 4.5. Antioxidant Activity

Antioxidant capacity was determined by the ABTS radical scavenging assay. Firstly, the radical cation ABTS^+∙^ was prepared by mixing 7 mM of ABTS (2,2′-azino-bis (3-ethylbenzothiazoline-6-sulphonic acid; Sigma-Aldrich) and 2.45 mM of potassium persulfate (K_2_S_2_O_8_) in a sodium phosphate buffer solution (0.1 M, pH 6.5). The mixture was incubated in darkness at room temperature for 16 h to facilitate the complete formation of the radical. For the reaction, the ABTS^+∙^ was diluted with phosphate buffet until absorbance levels at 727 nm were 0.75 ± 0.05. The assay was performed by adding together 950 μL of the diluted ABTS^+∙^ solution and 50 μL of algal extract, according with the methods of [106]. The samples were shaken, and absorbance was recorded by a UV–visible spectrophotometer at 727 nm at the beginning of the reaction (DOi) and after 8 min of incubation (DOf). The percentage of antioxidant activity (AA%) was calculated according to the following formula:

AA% = [(absDOi − absDOf)/absDOi] × 100 (1)

The concentration of antioxidant compounds was calculated using a standard curve of Trolox (6-hydroxy-2,5,7,8-tetramethylchroman-2-carboxylic acid) (Sigma-Aldrich) (20 to 100 µg·mL^−1^ − R^2^ = 0.99; y = 15.8928x; where y represents absorbance and x represents concentration), and the results expressed in μM of Trolox-equivalent antioxidant capacity (TEAC) per g of DW.

### 4.6. Statistical Analysis 

A Design of Experiments (DOE) methodology employing Box–Behnken designs was used to evaluate the effects of three independent factors (time, temperature, and enzyme concentration) on the extraction of soluble proteins through enzymatic hydrolysis. The experimental conditions were established using a Central Composite Design (CCD) combined with Response Surface Methodology (RSM), utilizing Statistica 10^®^ software (StatSoft, Tulsa, OK, USA). The factors and their levels under study were time (30, 105, and 180 min), temperature (20, 40, and 60 °C), and enzyme concentration (15, 30, and 45 mg of enzyme per g of dry biomass (mg·g^−1^)) (Supplementary Table S1), each with three replicates. 

The data passed the Shapiro–Wilk normality test and the Bartlett test for homogeneity of variance, and all samples were within the normal range and exhibited homoscedasticity. Alkaline hydrolysis data were analyzed using bifactorial analysis of variance (ANOVA) followed by Tukey’s a posteriori test. The two independent factors considered in the analysis were SC concentration (0%, 1%, 2.5%, 5.25%, and 10.5%) and temperature (45 °C and 80 °C), with the significance level set at *p* ≤ 0.05. Enzymatic hydrolysis data were analyzed using independent-samples *t*-tests (*p* ≤ 0.05). Subsequently, independent-samples *t*-tests (*p* ≤ 0.05) were also employed to evaluate the best treatments derived from each hydrolysis test. All statistical analyses were performed using the Statistica software package (Release 10.0). Additionally, the data were assessed using principal component analysis (PCA). Graphical representations were produced using scripts written in the Python language through the Spyder 5.5.5 software, in order to determine the similarities of physiological variables analyzed in the present study.

## 5. Conclusions

It is therefore concluded that the enzymatic hydrolysis significantly improves the extraction efficiency of most BACs in P. linearis, and that, for future applications, it would be beneficial to explore new combinations of temperature, time, and enzyme load to optimize the extraction of the compounds that did not achieve high efficiency levels in this study (phenols and MAAs). The substantial increase of BACs achieved by using enzymatic hydrolysis could be of interest to the nutraceutical industry, and especially with respect to MAAs in the cosmeceutical industry, which are used for both topical and oral UV-photoprotectors and considered to be more friendly to the environment [57]. Furthermore, if ingested, the commercial enzymes present in the extract assist in the human digestive process by breaking down the cell walls of ingested plants/vegetables, contributing to the absorption of nutrients.

## Figures and Tables

**Table 1:** Concentrations of phycobiliproteins (PE: phycoerythrin, PC: phycocyanin) (mg·g^−1^ dry weight) in *P. linearis* after extractions performed with alkaline hydrolysis (1%, 2.5%, 5.25%, and 10.5%) at 45 °C and 80 °C, and after extractions performed with enzymatic hydrolysis (control and ‘Miura’ cocktail) (*n* = 3; mean ± SD). In the samples from alkaline hydrolysis, different letters indicate significant differences according to the two-way analysis of variance and Tukey’s test (*p* ≤ 0.05). In the samples from enzymatic hydrolysis, asterisks indicate significant differences according to the independent *t*-test analysis (*p* ≤ 0.05).

Treatments	PE (mg·g^−1^DW)	PC (mg·g^−1^DW)
Water; 45 °C	4.44 ± 0.53 ^a^	0.75 ± 0.08 ^bcd^
Water; 80 °C	3.56 ± 0.34 ^abc^	0.64 ± 0.12 ^bcd^
Alkaline Hydrolysis (1%); 45 °C	3.04 ± 0.16 ^bcd^	0.73 ± 0.02 ^bcd^
Alkaline Hydrolysis (1%); 80 °C	3.64 ± 0.96 ^abc^	0.87 ± 0.17 ^abc^
Alkaline Hydrolysis (2.5%); 45 °C	2.39 ± 0.25 ^cde^	0.59 ± 0.03 ^cd^
Alkaline Hydrolysis (2.5%); 80 °C	4.12 ± 0.45 ^ab^	1.10 ± 0.15 ^ab^
Alkaline Hydrolysis (5.25%); 45 °C	1.32 ± 0.03 ^ef^	0.37 ± 0.04 ^d^
Alkaline Hydrolysis (5.25%); 80 °C	4.29 ± 0.54 ^ab^	1.24 ± 0.24 ^a^
Alkaline Hydrolysis (10.5%); 45 °C	0.78 ± 0.37 ^f^	0.27 ± 0.17 ^d^
Alkaline Hydrolysis (10.5%); 80 °C	1.73 ± 0.06 ^def^	0.66 ± 0.34 ^bcd^
Enzymatic Hydrolysis; Control	3.67 ± 1.76	3.26 ± 1.19
Enzymatic Hydrolysis; Miura	12.20 ± 3.70 *	6.71 ± 1.69 *

**Table 2:** Concentrations of total soluble phenolics, proteins, and carbohydrates (mg·g^−1^ dry weight) in *P. linearis* after extractions performed with alkaline hydrolysis (1%, 2.5%, 5.25%, and 10.5%) at 45 °C and 80 °C, and after extractions performed with enzymatic hydrolysis (control and ‘Miura’ cocktail) (*n* = 3; mean ± SD). In the samples from alkaline hydrolysis, different letters indicate significant differences according to the two-way analysis of variance and Tukey’s test (*p* ≤ 0.05). In the samples from enzymatic hydrolysis, asterisks indicate significant differences according to the independent *t*-test analysis (*p* ≤ 0.05).

Treatments	Phenols (mg·g^−1^DW)	Proteins (mg·g^−1^DW)	Carbohydrates (mg·g^−1^DW)
Water; 45 °C	1.37 ± 0.07 ^bc^	3.57 ± 0.40 ^cd^	40.35 ± 7.98 ^e^
Water; 80 °C	2.10 ± 0.19 ^b^	1.39 ± 0.16 ^f^	49.25 ± 2.63 ^de^
Alkaline Hydrolysis (1%); 45 °C	1.09 ± 0.40 ^c^	2.47 ± 0.61 ^ef^	69.48 ± 8.40 ^c^
Alkaline Hydrolysis (1%); 80 °C	3.19 ± 0.41 ^a^	4.91 ± 0.24 ^ab^	65.07 ± 7.47 ^cd^
Alkaline Hydrolysis (2.5%); 45 °C	1.71 ± 0.47 ^bc^	2.91 ± 0.57 ^de^	56.52 ± 7.20 ^cde^
Alkaline Hydrolysis (2.5%); 80 °C	3.53 ± 0.64 ^a^	5.63 ± 0.40 ^a^	116.50 ± 5.98 ^a^
Alkaline Hydrolysis (5.25%); 45 °C	1.61 ± 0.21 ^bc^	2.85 ± 0.25 ^de^	61.49 ± 8.73 ^cd^
Alkaline Hydrolysis (5.25%); 80 °C	2.09 ± 0.25 ^b^	4.47 ± 0.48 ^bc^	112.32 ± 6.41 ^a^
Alkaline Hydrolysis (10.5%); 45 °C	1.45 ± 0.10 ^bc^	2.02 ± 0.10 ^ef^	61.84 ± 7.07 ^cd^
Alkaline Hydrolysis (10.5%); 80 °C	1.97 ± 0.14 ^bc^	2.64 ± 0.19 ^de^	89.51 ± 7.53 ^b^
Enzymatic Hydrolysis; Control	1.81 ± 0.17	24.17 ± 6.12	87.97 ± 17.34
Enzymatic Hydrolysis; Miura	3.08 ± 0.22 *	55.63 ± 7.31 *	192.07 ± 24.60 *

**Table 3:** Concentrations of mycosporine-like amino acids (palythine, asterina-330, shinorine, porphyra-334, and total MAAs (sum of all identified MAAs)) (mg·g^−1^ dry weight) in *P. linearis* after extractions performed with alkaline hydrolysis (1%, 2.5%, 5.25%, and 10.5%) at 45 °C and 80 °C, and after extractions performed with enzymatic hydrolysis (control and ‘Miura’ cocktail) (*n* = 3; mean ± SD). In the samples from alkaline hydrolysis, different letters indicate significant differences according to the two-way analysis of variance and Tukey’s test (*p* ≤ 0.05). In the samples from enzymatic hydrolysis, asterisks indicate significant differences according to the independent *t*-test analysis (*p* ≤ 0.05).

Treatments	Palythine (mg·g^−1^DW)	Asterina-330 (mg·g^−1^DW)	Shinorine (mg·g^−1^DW)	Porphyra-334 (mg·g^−1^DW)	Total MAAs (mg·g^−1^DW)
Water; 45 °C	-	-	0.92 ± 0.04 ^a^	14.13 ± 1.18 ^b^	15.05 ± 1.22 ^b^
Water; 80 °C	0.010 ± 0.0001 ^a^	-	1.20 ± 0.31 ^a^	19.04 ± 4.48 ^a^	20.25 ± 4.80 ^a^
Alkaline Hydrolysis (1%); 45 °C	-	-	0.41 ± 0.14 ^b^	10.47 ± 0.85 ^bc^	10.88 ± 0.62 ^bc^
Alkaline Hydrolysis (1%); 80 °C	-	-	-	0.24 ± 0.09 ^e^	0.24 ± 0.08 ^e^
Alkaline Hydrolysis (2.5%); 45 °C	0.013 ± 0.004 ^a^	0.013 ± 0.003 ^a^	0.11 ± 0.05 ^bc^	6.36 ± 0.10 ^cd^	6.50 ± 0.15 ^cd^
Alkaline Hydrolysis (2.5%); 80 °C	-	-	-	-	-
Alkaline Hydrolysis (5.25%); 45 °C	-	-	0.07 ± 0.03 ^bc^	3.91 ± 0.29 ^de^	3.98 ± 0.31 ^de^
Alkaline Hydrolysis (5.25%); 80 °C	-	-	-	-	-
Alkaline Hydrolysis (10.5%); 45 °C	-	-	0.03 ± 0.01 ^c^	1.63 ± 0.26 ^e^	1.66 ± 0.94 ^e^
Alkaline Hydrolysis (10.5%); 80 °C	-	-	-	-	-
Enzymatic Hydrolysis; Control	-	-	-	15.85 ± 2.12	15.85 ± 2.12
Enzymatic Hydrolysis; Miura	-	-	-	20.75 ± 0.05 *	20.75 ± 0.05 *

**Table 4:** Concentrations of antioxidant agents (μmol of Trolox (TEAC)·g^−1^ dry weight) in *P. linearis* after extractions performed with alkaline hydrolysis (1%, 2.5%, 5.25%, and 10.5%) at 45 °C and 80 °C, and after extractions performed with enzymatic hydrolysis (control and ‘Miura’ cocktail) (*n* = 3; mean ± SD). In the samples from alkaline hydrolysis, different letters indicate significant differences according to the two-way analysis of variance and Tukey’s test (*p* ≤ 0.05). In the samples from enzymatic hydrolysis, asterisks indicate significant differences according to the independent *t*-test analysis (*p* ≤ 0.05).

Treatments	ABTS (µmol TEAC·g^−1^ DW)
Water; 45 °C	31.26 ± 4.09 ^ef^
Water; 80 °C	23.37 ± 1.68 ^f^
Alkaline Hydrolysis (1%); 45 °C	72.64 ± 6.15 ^e^
Alkaline Hydrolysis (1%); 80 °C	208.31 ± 13.62 ^ab^
Alkaline Hydrolysis (2.5%); 45 °C	123.63 ± 16.68 ^d^
Alkaline Hydrolysis (2.5%); 80 °C	236.85 ± 31.26 ^a^
Alkaline Hydrolysis (5.25%); 45 °C	150.02 ± 18.81 ^cd^
Alkaline Hydrolysis (5.25%); 80 °C	182.68 ± 16.79 ^bc^
Alkaline Hydrolysis (10.5%); 45 °C	164.33 ± 8.27 ^cd^
Alkaline Hydrolysis (10.5%); 80 °C	222.4 ± 9.22 ^ab^
Enzymatic Hydrolysis; Control	45.62 ± 5.61
Enzymatic Hydrolysis; Miura	61.45 ± 7.39 *

**Table 5:** Concentrations of phycobiliproteins (PE: phycoerythrin, PC: phycocyanin), phenolics, proteins, carbohydrates (mg·g^−1^ dry weight), and antioxidant agents (μM of Trolox (TEAC)·g^−1^ dry weight) in *P. linearis* following the optimal extraction performed with alkaline hydrolysis (2.5% sodium carbonate at 80 °C), and the best extraction performed with enzymatic hydrolysis (‘Miura’ cocktail) (*n* = 3; mean ± SD). Concentrations of total MAAs (mg·g^−1^ dry weight) in *P. linearis* after the optimal extraction with alkaline hydrolysis test (water-only at 80 °C), and the best extraction performed with enzymatic hydrolysis (‘Miura’ cocktail). Asterisks indicate significant differences according to the independent *t*-test analysis (*p* ≤ 0.05).

Treatments	PE (mg·g^−1^DW)	PC (mg·g^−1^DW)	Phenols (mg·g^−1^DW)	Proteins (mg·g^−1^DW)	Carbohydrates (mg·g^−1^DW)	ABTS (µM TEAC·g^−1^DW)	Total MAAs (mg·g^−1^DW)	Treatments
Alkaline Hydrolysis (2.5%); 80 °C	4.12 ± 0.45	1.10 ± 0.15	3.53 ± 0.64	5.63 ± 0.40	116.50 ± 5.98	236.85 ± 31.26 *	20.25 ± 4.80	Water; 80 °C
Enzymatic Hydrolysis; Miura	12.20 ± 3.70 *	6.71 ± 1.69 *	3.08 ± 0.22	55.63 ± 7.31 *	192.07 ± 24.60 *	61.45 ± 7.39	20.75 ± 0.05	Enzymatic Hydrolysis; Miura

## Data Availability

Data is available upon request.

## Supplementary Materials

The following supporting information can be downloaded at: https://www.mdpi.com/article/10.3390/md22060284/s1. Table S1. DoE matrix generated from the minimum, medium, and maximum points of temperature, time, and enzymatic concentration, with the content levels of soluble proteins obtained) (*n* = 3; mean ± SD).
