# Supplementary material for: Optimizing the Extraction of Bioactive Compounds from Porphyra linearis (Rhodophyta): Evaluating Alkaline and Enzymatic Hydrolysis for Nutraceutical Applications
Source: Mar Drugs. 2024 Jun 18;22(6):284. doi: 10.3390/md22060284 (PMC11204741; doi:10.3390/md22060284)
Supplement: Supplementary file 1 [file marinedrugs-22-00284-s001.zip › marinedrugs-3055493-supplementary.pdf]

Supplementary Table

Table S1. DoE matrix generated from the minimum, medium, and maximum points of temperature, time, and enzymatic concentration, with the soluble proteins content obtained (*n* = 3; mean ± SD).

Treatment	Temperature (°C)	Time (min)	Enzyme Concentration ($mg{\cdot}g^{-1}$)	Soluble Protein ($mg{\cdot}g^{-1}$)
**1**	20	30	30	22.16 ± 2.39
**2**	60	30	30	6.00 ± 2.32
**3**	20	180	30	25.14 ± 1.97
**4**	60	180	30	-
**5**	20	105	15	26.52 ± 3.66
**6**	60	105	15	6.49 ± 1.01
**7**	20	105	45	33.41 ± 5.20
**8**	60	105	45	-
**9**	40	30	15	19.51 ± 1.34
**10**	40	180	15	23.24 ± 2.95
**11**	40	30	45	14.22 ± 2.82
**12**	40	180	45	28.69 ± 1.90
**13**	40	105	30	32.40 ± 7.17
